# The Use of Antimicrobials in Animal Husbandry as a Potential Factor for the Increased Incidence of Colorectal Cancer: Food Safety and Kinetics in a Murine Model

**DOI:** 10.3390/ani15030315

**Published:** 2025-01-23

**Authors:** Rosa D’Ambrosio, Stefania Cavallo, Roberta Brunetti, Roberta Pellicanò, Emanuela Vaccaro, Giorgia Borriello, Rubina Paradiso, Francesco Paolo Serpe, Sara Lambiase, Francesca Bruzzese, Giuseppe Palma, Domenica Rea, Antonio Barbieri, Marianna D’Amore, Maria Dimatteo, Barbara degli Uberti, Orlando Paciello, Loredana Baldi

**Affiliations:** 1 Istituto Zooprofilattico Sperimentale del Mezzogiorno (IZSM), 80055 Portici, Italy; rosa.dambrosio@izsmportici.it (R.D.); stefania.cavallo@izsmportici.it (S.C.); roberta.pellicano@izsmportici.it (R.P.); giorgia.borriello@izsmportici.it (G.B.); rubina.paradiso@izsmportici.it (R.P.); francesco.serpe@izsmportici.it (F.P.S.); sara.lambiase@izsmportici.it (S.L.); marianna.damore@izsmportici.it (M.D.); barbara.degliuberti@izsmportici.it (B.d.U.); loredana.baldi@izsmportici.it (L.B.); 2 Department of Veterinary Medicine and Animal Production, University of Naples Federico II, 80137 Napoli, Italy; emanuelavaccaro3@gmail.com (E.V.); paciello@unina.it (O.P.); 3 Experimental Animal Unit, Istituto Nazionale Tumori-IRCCS-Fondazione “G. Pascale”, 80131 Naples, Italy; f.bruzzese@istitutotumori.na.it (F.B.); giuseppe.palma@istitutotumori.na.it (G.P.); 4 Laboratory Medicine Unit, Istituto Nazionale Tumori- IRCCS- Fondazione “G. Pascale”, 80131 Naples, Italy; d.rea@istitutotumori.na.it; 5 ASL Salerno UOC Laboratorio d’Analisi, Vallo della Lucania, 84078 Salerno, Italy

**Keywords:** antimicrobials, colorectal cancer, One Health, food safety

## Abstract

The aim of this research was to investigate the effects of the prolonged use of the broad-spectrum antimicrobial widely used in animal husbandry. By means of a mouse model, a translational study was carried out on immunocompetent mice (with a complete immune system). This study highlighted the effect of antimicrobial residues taken in with food on the growth time of cancer and on alterations to the gut microbiota. This project considered the fight against antimicrobial resistance from a One Health perspectivethrough collaboration between human medicine and veterinary medicine. Regarding food safety, antimicrobial residues in products of animal origin are rarely detected; they therefore constitute a negligible factor in determining colorectal cancer.

## Simple Summary

This project aimed to investigate the effects of prolonged administration of the broad-spectrum antimicrobials widely used in animal husbandry. By generating an animal engraftment model, it was assessed whether the accumulation of these antimicrobials or their residues over time could constitute a risk for consumers of animal-derived foods. In order to improve knowledge of the onset of colorectal cancer, the present study evaluated how antimicrobials could affect the composition of the gut microbiome and the immune system.

## 1. Introduction

The fight against antimicrobial resistance is one of the primary objectives of the Single Medicine (One Health) approach, which is an integrated, unifying approach that aims to sustainably balance and optimize the health of people, animals and ecosystems; it recognizes that the health of humans, domestic and wild animals, plants, and the wider environment (including ecosystems) are closely linked and interdependent [1]. In the context of food Safety, this strategic goal is pursued through the implementation of various sampling plans, including the National Residue Plan (PNR) [2]. The term “residues” refers to the molecules accumulated in meat, milk, eggs, or other products derived from animals treated with antimicrobials.

To safeguard public health and limit the presence of antimicrobials in food products, safety limits have been established for human consumption [2,3]. Despite these measures, the accumulation of these residues due to prolonged consumption of animal products may pose several risks to human health, including allergic reactions, the development of antimicrobial resistance, carcinogenesis, mutagenesis, teratogenesis, and the destruction of normal intestinal flora.

It is known that antimicrobials can induce changes in the composition of the gut microbiota, leading to chronic dysbiosis and the onset of precancerous lesions. Antimicrobial residues may also impact the function of the immune system, causing alterations that can promote tumor development. A study conducted on women aged 20 to 39 years who were treated with antimicrobials for at least two months showed a 36% higher probability of being diagnosed with colorectal adenoma than untreated subjects [4]. Another American study, called the “Nurses’ Health Study”, investigated the lifestyles and dietary habits of approximately 20,000 health professionals [5] and revealed an increased risk of developing colorectal cancer among individuals who frequently took antimicrobials. In 2018, the Italian Istituto Superiore di Sanità conducted a survey on population exposure to antimicrobials through the consumption of animal-derived foods. This study identified sulfonamides and tetracyclines as target molecules [2].

The development of antimicrobials is considered one of the greatest medical advances of the 20th century [6]. However, worldwide antimicrobial use increased by 65% between 2000 and 2015 [7]. Although most courses of antimicrobial treatment have no apparent adverse effects, antimicrobials can cause significant changes in the gut microbiota, giving rise to both short- and long-term health consequences [8]. Observational studies have also implicated the use of antimicrobials in the pathogenesis of other increasingly prevalent conditions, including gastrointestinal infections, weight gain and obesity, inflammatory bowel disease (IBD), and colorectal cancer [9]. Other serious consequences of the use of antimicrobials are the development of bacterial resistance to antimicrobials [10] and alteration to the functions of the microbiota, producing long-lasting deleterious effects in the host.

The influence of the intestinal microbiota on the health of the host is known [11]. Indeed, experimental and clinical evidence has shown that intestinal microorganisms are necessary for the optimal functioning of the host [12]; the gut microbiota plays a fundamental role in many physiological and pathological processes, and the efficacy of some clinical approaches is also conditioned by the action of commensal bacteria. The “microbiota” is defined as the set of microorganisms present in a particular environment, while the term “microbiome” refers to the entire habitat, including microorganisms (bacteria, Archaea, eukaryotes, and viruses), their genomes, and the environmental conditions that characterize the specific habitat [13,14].

The development of culture-independent research methods that combine genetic sequencing with bioinformatics has led to rapid advances in the study of the gut microbiota [12]. One of the most common methods used for the taxonomic identification and diversity assessment of prokaryotic species (Bacteria and Archaea) is the sequencing of the gene encoding the small subunit of ribosomal RNA (16S rRNA) [12]. Most of the strains that make up the gut microbiota reside stably in the gut, although their relative abundance varies over time [12]. However, longitudinal studies have indicated that numerous external factors, such as diet, substance intake, lifestyle, disease, or colonic transit time, affect the composition of the gut microbiota [15,16,17]. Although intra-individual changes in the composition of the gut microbiota are significant, the bacterial community remains quite stable over time, always returning to the condition it was in before the intervention of external factors—a quality known as resilience [15,16]. The diversity of the gut microbiota also changes with age. Gut microbial changes correlate with nutritional status and the overall level of inflammation.

In the present study, in vivo experimentation was carried out on immunocompetent mice, in order to evaluate the effects of exposure to antimicrobial residues taken in with food on both the gut microbiota and tumor progression. This project aims to investigate the effects of prolonged exposure to the broad-spectrum antimicrobials widely used in the animal husbandry sector. Having constructed an animal engraftment model, we evaluated whether the accumulation of antimicrobials or their residues over time could constitute a danger by relating our data to the consumption of foods of animal origin.

## 2. Materials and Methods

### 2.1. Identification of the Molecules of Interest

The molecules of interest were identified by the Istituto Zooprofilattico Sperimentale del Mezzogiorno (IZSM) by using data from samples taken in the Campania region for the National Residues Plan (PNR) in the period 2015/2018.

The PNR is a surveillance plan that has been implemented in Italy for many years to search for residues of pharmacologically active substances in live animals, tissues, and food of animal origin. Sampling can be carried out both on the farm and in primary processing plants of products of animal origin [2]. Since no excessive values were found for the matrices covered by the plan, we used data on antimicrobials with values higher than the limit of quantification (LOQ); this led to the identification of tetracyclines and sulfonamides as families of antimicrobials of interest. In particular, data from follow-up analyses, reported in the introduction section of the National Residue Plan, implemented over the last years allowed for identifying the antimicrobial molecules most widely used and most frequently found as active ingredients and as residues in foods of animal origin; muscle, milk, liver, and egg samples were considered.

Tetracyclines are widely used, owing to their low cost and broad spectrum of action. Regarding the toxicity of these substances, it has been demonstrated that exposure to a single dose of tetracyclines in quantities higher than the acceptable daily dose (ADI) has a negative impact on the proliferation of intestinal epithelial cells, compromises the integrity of the intestinal wall, allows for the translocation of bacteria, and reduces the expression of several genes that are essential for wall integrity [18,19]. Moreover, adverse effects of tetracyclines in terms of interference in the hematopoiesis process have been ascertained. Concerning sulfonamides, the other category of widely used antimicrobials considered, there is clear evidence of hypersensitivity reactions following treatment [20]. In this case, too, disorders of the hematopoietic system, intestine, nerves, and liver have been documented.

### 2.2. Evaluation of Exposure Level

The antimicrobials under study were administered to groups of mice at doses comparable to a standard human dose assumed via food intake with concentrations equal to the regulatory limit. In particular, after one week of quarantine, the mice (21 male and 21 female) were divided in three different experimental groups, control, sulfonamides, and tetracyclines (7 mice/group), to receive for 4 weeks the antibiotics. Mice in the control groups were treated with the vehicle in which the antibiotics were dissolved. At the end of 4 weeks, mice were inoculated subcutaneously into the flank regions with 1 × 10^6^ of CT26 cells resuspended in 200 μL of PBS. Antibiotics treatment continued for another 2 weeks, during tumor growth.

To calculate the standard dose, the following scheme was applied:

A. Selections of foods for which there is a regulatory limit for sulfonamides and tetracyclines (Table 1);
B. Calculation of daily requirements in terms of major nutrients (carbohydrates, fats, and proteins) expressed in g/day for a 30-year-old adult individual;
C. Identification of the amount of food of animal origin corresponding to the daily requirement;
D. Definition of a standard meal in order to calculate the intake of sulfonamides and tetracyclines.

As opposed to a complete diet, we considered only foods of animal origin with a regulatory limit for sulfonamides and tetracyclines, as established by Commission Regulation (EU) N^o^. 37/2010 of 22 December 2009 on pharmacologically active substances and their classification (in Consolidated Text form of 29 September 2018), assuming the intake of foods with concentrations equal to the regulatory limit. Table 1 shows the maximum limit allowed by Regulation (EU) No. 37/2010 regarding the families of antimicrobials considered and the reference matrices.

A. Regarding all food matrices of animal origin in EU Regulation 37/2010, we decided to exclude liver, fat, and kidney, as these are rarely used in human nutrition [21]; we therefore used the following matrices:
  - Muscle (from beef, pork, poultry, and fish);
  - Cow’s milk;
  - Hen eggs (for chlortetracycline only).
B. To calculate the daily requirements in terms of major nutrients (carbohydrates, fat, and protein) for a 60 kg adult, we used the interactive tool that the EFSA makes available online (DRV Finder) [22] and applied the dietary reference values (DRVs) established by the EFSA.
  In this study, the following parameters were adopted:
  - Thirty-year-old adults of both sexes;
  - Nutrients: protein;
  - Population Recommended Intake (PRI) values.
C. To trace the EFSA DRV Finder values for foods commonly used in the Italian diet, the Food Composition Tables Database available online from the CREA (Center for Research on Food and Nutrition) website was used; the standard reference quantity for a portion of food was set to 100 g and the following matrices were selected:
  - Adult beef or veal loin [muscle tissue stripped of visible fat];
  - Pork loin, raw;
  - Chicken breast, raw;
  - Cod or hake, raw;
  - Pasteurized cow’s milk, semi-skimmed;
  - Hen eggs, whole.
D. On the basis of these data, a typical day’s diet was drawn up for an adult weighing 60 kg; from these amounts of food, the intake of tetracyclines and sulfonamides was calculated, assuming that the foods contained concentrations of these antimicrobials equal to the regulatory limit (EU Regulation 37/2010).
E. For the mouse dosage calculation, the conversion from human to mouse was determined using the following equation [23]:

Human Equivalent Dose = Dose mouse × 3/37 (Km human/Km mouse) where Km is the conversion factor.

### 2.3. In Vitro Study

The colon adenocarcinoma cell line CT26 (American Type Culture Collection—ATCC, Rockville, MD, USA) was used in this study. Cells were cultured in Dulbecco’s modified Eagle’s medium supplemented with serum and glutamine. They were maintained at a temperature of 37 °C in a humidified atmosphere of 5% CO_2_ and used in in vitro assays designed to evaluate changes in cell proliferation in relation to the concentration of antimicrobials used. The following assays were carried out:

- Cell viability assay;
- Wound healing assay (48 h);
- Colony-forming assay (15 days).

In vitro studies used both tetracyclines and sulfonamides.

### 2.4. In Vivo Study

The use of an animal model is a key step when undertaking preclinical studies aimed at translational medicine. This project involved the development of syngeneic mouse models involving the implantation of 1×106 CT26 colon cancer cells in the subcutis of immunocompetent inbred Balb/c strain mice. Using inbred mice allowed for us to apply the principle of “reduction” of the 3 Rs model (Replacement, Reduction, Refinement) [24] to respect animal welfare. Since these animals are mated for 20 generations, their biological responses to treatments are almost identical; this allowed for us to use the minimum number of animals without sacrificing statistical power. All animal procedures were performed in accordance with ARRIVE guidelines [25] and were approved by the appropriate institutional review board (no. 343/2021-PR). In particular, the mice were acclimatized to the Animal Care Facility of INT-IRCCS “Fondazione G. Pascale”, Naples. The recruited animals (21 males and 21 females) were divided and randomized according to weight based on the random number generator function in Microsoft Excel, into 3 groups of 7 mice each, and subdivided as shown in Table 2.

Sample size was based on estimations by power analysis (G*Power version 3.1.9.7) with a level of significance α (probability error) of 0.05 and a power of 0.8 (1-β err prob) selecting the F tests as the test family and the ANOVA as the statistical fixed effects test, omnibus, one-way. The number of animals per group is also based on previous studies performed to induce gut microbiota dysbiosis [26,27,28].

An effect size f of 0.40 and a number of experimental groups of 3 per experiment (control, sulfonamide-treated, and tetracycline-treated) as default input parameters. Statistical analysis of the results obtained was conducted by means of a Student’s *t*-test among groups.

Animals were weighed and monitored for one month before subcutaneous tumor cell inoculation, and thereafter for the assessment of tumor rooting and growth. Humane endpoint cutoffs were defined as a tumor volume of 1500 mm^3^ and a 20% weight reduction.

When the mice were euthanized, the tumor samples were taken and placed in formalin for histological and immunohistochemical evaluations.

The dose of antimicrobial administered was calculated for a final volume of 200 mL dissolved in water, which was equal to the contents of the animals’ drinking bottles and changed every 3 days. This route of administration is preferable to oral gavage, as it minimizes the impact of stress on the animals, in compliance with the 3Rs: Replacement, Reduction, Refinement. Specifically, in the case of chlortetracycline, on the basis of a calculated daily intake of 0.84 µg/kg body weight via food in humans (see Results), the dose to be administered to each mouse was determined to be 10.4 µg/kg mouse body weight; this calculation was made according to the calculation Human Equivalent Dose = Mouse Dose × 3/37 (kg human/kg mouse). The same approach was adopted for sulfonamides: the conversion calculation predicted the sulfonamide dose to be 8.3 µg/kg mouse body weight. At the end of the 4-week pretreatment period, the heterotopic colorectal cancer model was generated with the CT-26 syngeneic line for the Balb/c mouse strain.

**Figure 1:**
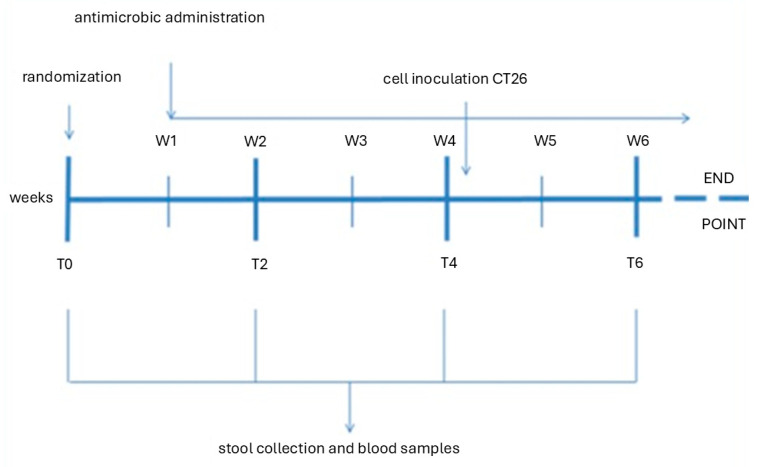
Time scheme of in vivo experiment.

Treatment with antimicrobials was continued for a further 2 weeks during the tumor growth phase. The size of the tumor masses was recorded biweekly by means of a digital caliper. During this experimental stage, stool and blood sampling were performed in week 6. When the experimentally ethical cut-off value had been reached (mass volume of 1.5 cm^3^), the mice were sacrificed (Figure 1).

### 2.5. Sampling for Microbiota Analysis

In order to characterize the gut microbiota of animals in the present study, fecal samples were collected with sterile tweezers at three different time points in all groups, in order to evaluate possible differences due to the administration of the different treatments. Samples were collected from each animal at three different time points: before the administration of the selected molecules (T0), after a month of treatment (T30), and upon sacrifice after two months of treatment (T60). The choice of these time points was made to consider the short-term variation (T30) and whether observed changes increased and became consolidated over a longer period (T60). Indeed, several studies have shown that gut microbial communities can undergo rapid changes in response to perturbations, such as antimicrobial use [29], and these changes may produce long-term effects on the gut microbiota [30]. Furthermore, it has been observed that differences between samples collected over short time intervals are significantly smaller than those observed over longer intervals, suggesting that short-term variation in gut microbiota tends to increase over time [31].

All samples were stored at −80 °C until further analysis.

### 2.6. DNA Extraction and Sequencing

DNA extraction from stool samples was performed according to the standard stool sample protocol IHMS_SOP 07 V2 Version 2 [32], with minor modifications owing to the use of the QIASymphony automated extractor and the QIAamp DSP DNA Mini Kit (Qiagen, Hilden, Germany). DNA extracted from the samples was evaluated by means of the QuibitTM 2.0 high-sensitivity fluorometer (Thermo Fisher Scientific, Waltham, MA, USA), in order to have a final concentration of 10 ng/µL for all samples. In addition, PBS was used for sample preparation as a negative control.

Sequencing of the V2, V3, V4, V6, V7, V8, and V9 hypervariable regions of the 16S rRNA gene was performed by using the primer sets from the Ion 16S Metagenomics kit (Thermo Fisher Scientific, MA, USA), the Ion S5 XL sequencer, and the Ion 530 chip.

Raw 16S rRNA reads were analyzed by means of QIIME 2-2019.7 software. All sequences were checked for quality in order to exclude those of poor quality (Phred score < 20), so as not to affect the downstream analysis. The dada2 package was used to perform de-noising, removal of primers and chimeras, and to exclude low-quality sequences. Good-quality sequences were then resolved into high-resolution Amplicon Sequence Variants (ASVs). ASVs with 99% similarity were collected by means of the uclust method [33]. The taxonomic assignment of each ASV was determined according to the Silva 138 database [34]. Finally, any change in microbiota composition and variability was assessed both for paired (t0 vs. t30 vs. t60) samples over time and for unpaired samples (control vs. sulfonamide vs. tetracycline) according to the different groups, by using alpha and beta diversity measures.

### 2.7. Histopathological Examination and Immunohistochemistry

Histological examination was performed on liver and subcutaneous tumor samples in 40 individuals (20 males and 20 females) divided into the following groups: 14 controls, 14 treated with tetracyclines, and 12 treated with sulfonamides. Samples were fixed in 10% neutral buffered formalin, trimmed, and embedded in paraffin. Microsections (3–4 μm thick) were routinely stained with hematoxylin and eosin. Histological examination of livers took into consideration degenerative process scored according to the histological grading of [35]. For immunohistochemistry (IHC), additional sections of the 40 subcutaneous tumor samples (3–4 µm thick) were processed with the Bond Polymer Refine Detection Kit (Leica Biosystem). The primary antibodies were CD4 and CD8, ready to use. As positive control tissue, sections of tonsils from the same animals were used for both antibodies. Skeletal muscle and cerebellum samples were used as negative control tissue [36]. The slides were automatically stained by means of Leica Bond III immunostainers. Slides were examined and photographed with a Pannoramic 250 Flash III slide scanner. In each case, cells were counted in 10 random fields of 0.7 mm^2^ at high magnification, in order to obtain a minimum and maximum mean value for each group assessed.

## 3. Results

### 3.1. Calculation of Dosages

Tetracycline and sulfonamide antimicrobials were the most frequently detected residues in food in the period 2015–2018 according to the “National Residue Plan” and “Targeted Investigations following Suspected Nonconformities”. Specifically, out of 407 determinations, molecules belonging to the tetracycline family were detectable in 25 cases. On the basis of these data, chlortetracycline was selected as the target molecule for the purposes of the present study. Similarly, from the determination of sulfonamide residues in 744 samples, the presence of sulfadimethoxine was detected in 21 samples.

On the basis of the PRI (Population Recommended Intake) values from the EFSA DRV Finder, the protein requirement of a 30-year-old adult is 0.83 g/kg b.w. per day, which is 0.83 × 60 = 49.8 g of protein per day for a 60 kg individual.

According to CREA data [37], the proteins contents per 100 g of edible parts of selected foods (Table 3) are:

- Adult bovine loin: 21.8 g of protein per 100 g of edible part;
- Pork loin: 20.7 g of protein per 100 g of edible part;
- Chicken breast: 23.3 g protein per 100 g edible part;
- Cod: 17 g protein per 100 g edible part;
- Milk: 3.5 g of protein per 100 g of edible part;
- Eggs: 12.4 g protein per 100 g edible part.

In a hypothetical typical diet that includes the consumption of milk, eggs, chicken breast, and fish in a day, the total protein intake corresponds to the values shown in Table 4.

The total protein intake (53.872 g) turns out to be slightly higher (+4.072 g) than the EFSA-recommended intake (49.8 g).

Hypothetically, in all matrices that have an antimicrobial residue equal to the regulatory limit, the dietary intake of these antimicrobials is that shown in Table 5.

For a 60 kg adult with a hypothetical diet containing residue values equal to the limit imposed by Reg. 37/2010, the amount of antimicrobials ingested in the diet is shown in Table 6.

On the basis of these values (0.84 µg/kg for chlortetracycline and 0.67 µg/kg for sulfonamides we converted a hypothetical human dietary intake of antimicrobials into the corresponding value to be administered to mice during the experimental phase of this study.

### 3.2. In Vitro Study

**Figure 2:**
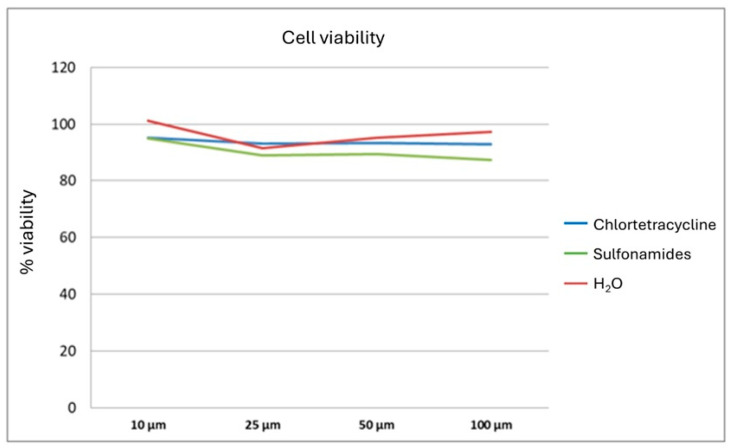
The in vitro experiments showed no significant effects of the antimicrobials on the CT26 cell line on viability assays.

**Figure 3:**
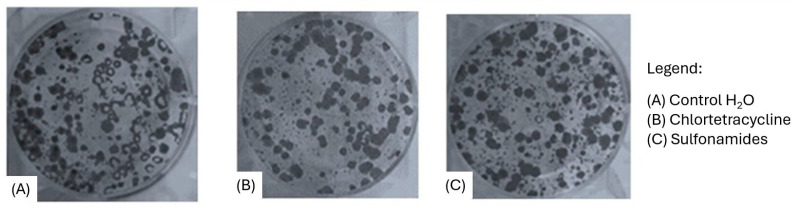
Colony formation assays.

The in vitro study involved the use of the colon adenocarcinoma cell line CT26 for viability, migration, and colony formation assays following treatment with the antimicrobials under study. The antimicrobials displayed no significant effects on CT26 cell viability, as assessed by means of MTT (Figure 2) and colony formation assays (Figure 3).

### 3.3. In Vivo Study

**Figure 4:**
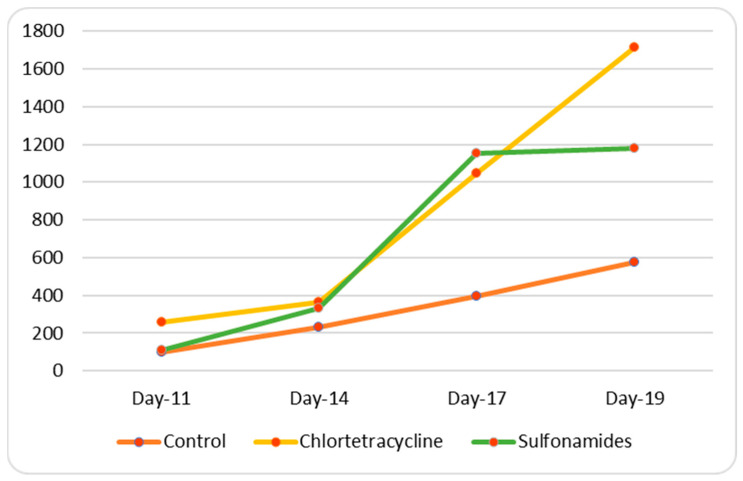
Trend in tumor growth in females.

**Figure 5:**
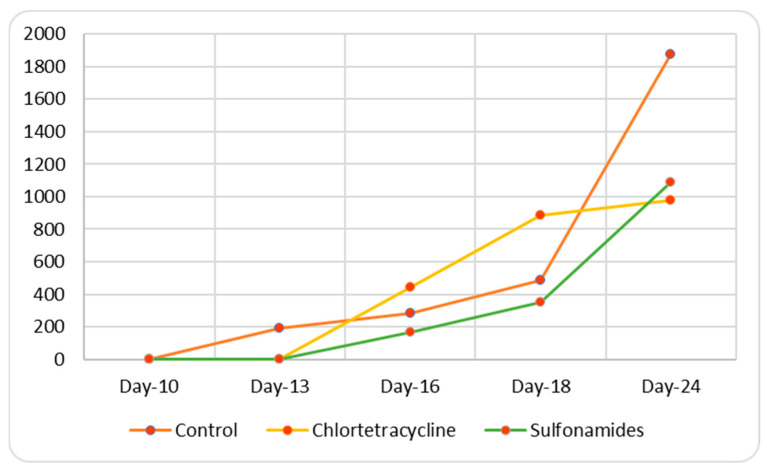
Time-course of tumor growth in males.

In contrast with the results obtained in vitro, the in vivo experiment revealed a difference in tumor cell rooting (Table 7 and Table 8) after inoculation in both males and females. Specifically, in the control group, greater rooting was found in females than in males, and a different pattern of tumor growth emerged over time between the two genders, as shown in Figure 4 and Figure 5. This finding is consistent with previous data [38].

Indeed, estrogens can exert a pleiotropic action, inducing cancer cells to secrete factors that enhance tumor growth and rooting. Conversely, tumor growth in antimicrobial-treated male mice was slower than in the corresponding group of females, indicating a different effect of antimicrobials according to sex.

Student’s *t*-test showed that in female mice, chlortetracycline treatment considerably increased tumor growth in comparison with the control group (*p*-value < 0.05 control vs. chlortetracycline), while non-significant differences were found after sulfonamide treatment (*p*-value > 0.05 control vs. sulfonamides). Finally, a statistically significant difference (*p*-value < 0.05 sulfonamides vs. chlortetracycline) was found on comparing the two treated groups, with chlortetracycline treatment promoting the growth of cancer cells.

These results confirm the correlation between hormonal aspects and the immune system. Indeed, the antimicrobials induced different effects in males and females.

**Figure 6:**
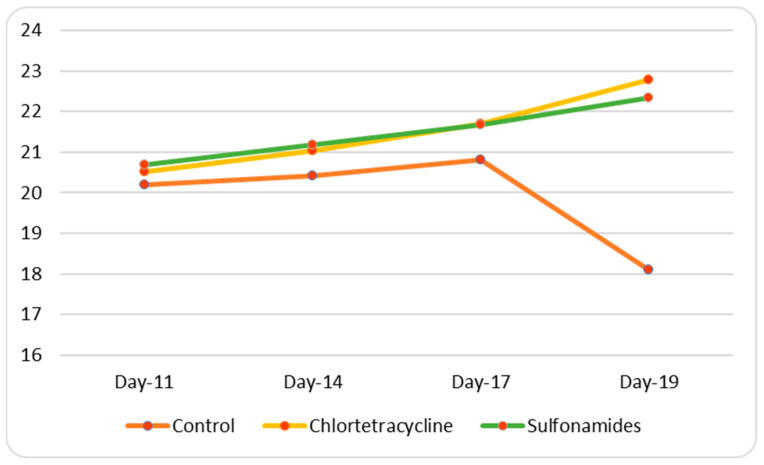
Weight trend in females.

**Figure 7:**
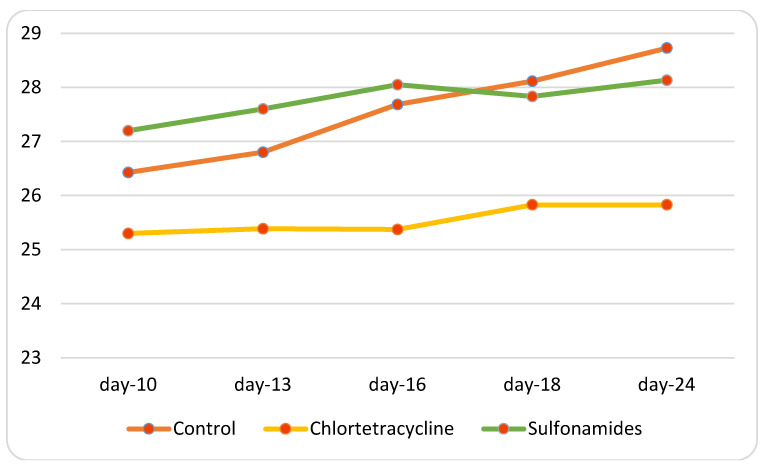
Weight trend in males.

No significant weight loss attributable to antimicrobial toxicity was observed in the treatment groups when compared with the control groups (Figure 6 and Figure 7).

### 3.4. Sequencing Analysis and Microbiota Characterization

**Figure 8:**
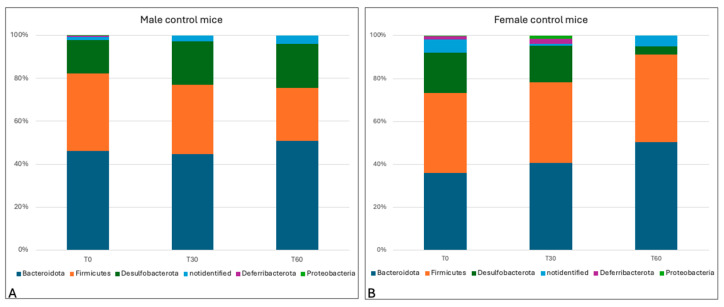
Taxa bar plots showing the relative frequencies of the phyla in male control mice (not treated with antimicrobials) (**A**) and in female control mice (**B**), at the different experimental times (0, 30, and 60 days).

Sequencing produced a total of 2,386,013 reads, with a mean number of 159,067 sequences per sample used for compositional and statistical analysis. In the present study, we first evaluated the taxonomic composition of the microbiota in control groups; this showed the presence of three main phyla. The relative abundances in the male group (mean > 1%) highlighted the presence of *Bacteroidota* (47.28% ± st.dev. 2.7%), *Firmicutes* (48.1% ± 4.8%), and *Desulfobacterota* (18.82% ± 2.38%), while the female group showed the presence of *Bacteroidota* (42.41% ± st.dev. 7.34%), *Firmicutes* (38.51% ± 2.02%), and *Desulfobacterota* (13.24% ± 8.2%). No substantial differences emerged between the groups under study, since the frequencies were similar in both sexes; this similarity was preserved, thus indicating the stability of the phyla over time (Figure 8).

**Figure 9:**
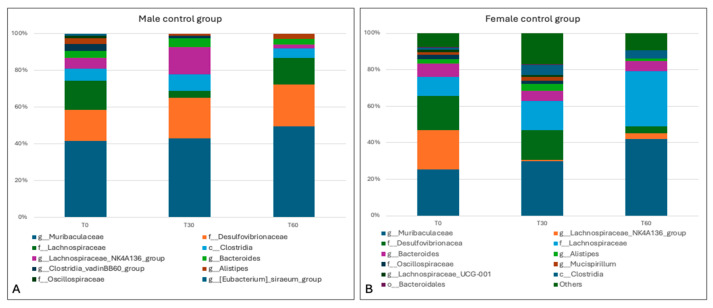
Taxa bar plots showing the relative frequencies of the genera identified in male control mice (not treated with antimicrobials) (**A**) and in female control mice (**B**), at the different experimental times (0, 30, and 60 days).

A total of 97 genera were identified. In the male group, the most frequently identified taxa (i.e., with a relative abundance >1%) were *Muribaculaceae* (40.67% ± 2.89%), unclassified genus of the family *Desulfovibrionaceae* (18.33% ± 2.89%), unclassified genus of the family *Lachnospiraceae* (10.67% ± 5.86%), *Lachnospiraceae*_NK4A136_group (7% ± 6.24), unclassified genus of the class *Clostridia* (6% ± 2%), *Bacteroides* (3.67% ± 0.58%), unclassified genus of the family *Oscillospiraceae* (2% ± 2.65%), *Alistipes* (2.33% ± 1.15%), and *Clostridia_vadin* BB60_group (1.33% ± 1.53%). In the female group, the relative frequencies were *Muribaculaceae* (32.33% ± 8.74%), unclassified genus of the family *Lachnospiraceae* (19% ± 9.85%), unclassified genus of the family *Desulfovibrionaceae* (13% ± 7.94%), *Lachnospiraceae_NK4A136_group* (8.67% ± 11.59%), *Bacteroides* (6% ± 1%), unclassified genus of the class *Clostridia* (3.67% ± 2.31%), *Alistipes* (2.67% ± 1.53%), *Clostridia_vadinBB60_group* (1.67% ± 2.89%), unclassified genus of the family *Oscillospiraceae* (1.33% ± 1.15%), and *Mucispirillum* (1.33% ± 1.15%) (Figure 9).

The relative frequencies of the genera identified in the control groups showed some differences in the gut microflora, probably due to the natural physiological evolution of the microbiota and partly to the sex of the study subjects. However, statistical analysis, performed using the Wilcoxon signed-rank test for paired and Mann–Witney U test for unpaired samples, did not show significant differences in beta diversity (*p* value > 0.05), consistent with the data previously displayed for the phyla identified (Supplementary Figures S1 and S2).

### 3.5. Characterization of the Effects of Antimicrobials on the Gut Microbiota

Analysis of the composition of the gut microbiota in the treated groups showed a significant influence of the two classes of antimicrobials under study. In particular, treatment with tetracyclines displayed a much stronger impact on the microbiota structure than sulfonamides, in terms of both species richness and relative abundance.

The mean relative frequencies in male mice treated with sulfonamides showed a decrease in *Firmicutes* (35.6% ± 11.88%) and *Desulfobacterota* (8.15% ± 1.48%) and an increase in *Bacteroidetes* (53.35% ± 11.95%), resulting in a decreased F/B ratio compared with the control group.

**Figure 10:**
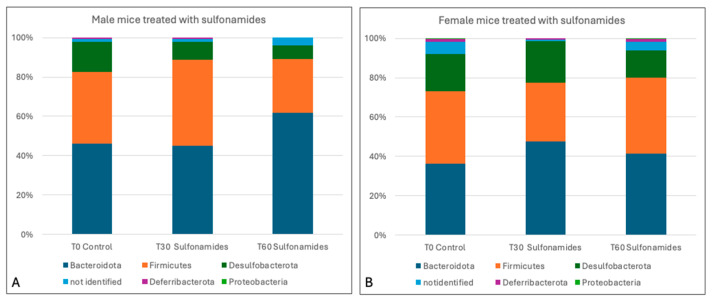
(**A**) Taxa bar plot showing the relative abundance of the phyla identified in male (**A**) and female mice (**B**) treated with sulfonamides at the different experimental times (0, 30, and 60 days).

Consistently, in female mice, the data obtained indicated a decrease in *Desulfobacterota* (17.5% ± 4.81%), with a constant *Firmicutes/Bacteroidota* ratio (Figure 10).

**Figure 11:**
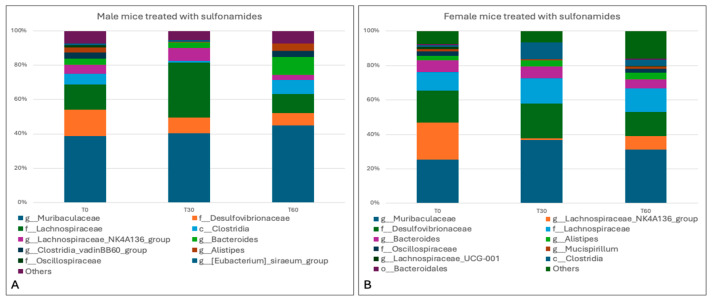
Taxa bar plot showing the relative abundance of the genera identified in male (**A**) and female mice (**B**) treated with sulfonamides at the different experimental times (0, 30, and 60 days).

In keeping with the data reported above, several genera in sulfonamide-treated male mice showed an increased abundance of specific *Bacteroidetes* species, i.e., *Muribaculaceae* (42.6% ± 3.25%) and *Bacteroides* (6.8% ± 5.09%), and a decrease in *Firmicutes* species *Clostridia_vadin BB60_group* (1.85% ± 2.62%) and *Eubacterium* (0.5% ± 0.71%), thus altering the F/B ratio. In sulfonamide-treated female mice, by contrast, the observed variation in relative frequencies of the genera of the *Bacteroidetes* and *Firmicutes* phyla did not affect the F/B ratio. Indeed, we observed increases in *Muribaculaceae* (34.05% ± 4.03%) and *Alistipes* (3.75% ± 0.21%), which were balanced by a decrease in *Bacteroides* (6% ± 1.13%), and increases in *Lachnospiraceae* (14.15% ± 0.49%) and *Clostridia* (6.65% ± 4.6%), which were balanced by decreases in *Oscillospiraceae* (1.15% ± 1.63%) and *Lachnospiraceae_NK4A136_group* (4.45% ± 5.02%) (Figure 11). Treatment with sulfonamides was less well tolerated by female mice, which exhibited a significant variation in the relative frequency of the *Muribaculaceae* family (*p*-value < 0.05).

**Figure 12:**
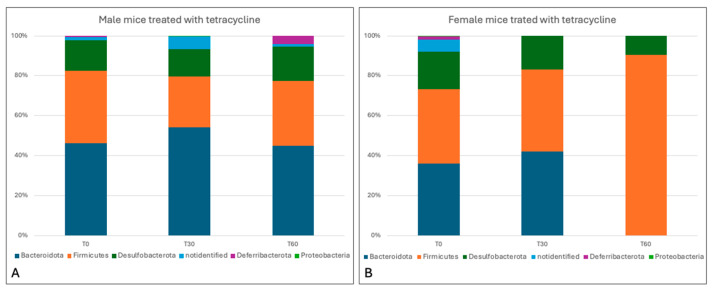
Taxa bar plot showing the relative abundance of the phyla identified in male (**A**) and female mice (**B**) treated with tetracycline at the different experimental times (0, 30, and 60 days).

Different results were obtained in tetracycline-treated mice, with a greater impact on the intestinal microbiota being observed in both gender groups, particularly in the female group. Indeed, the results revealed a marked decrease in *Firmicutes* (28.9% ± 5.09%) and increases in *Bacteriodetes* (49.6 ± 6.65%) and *Deferribacterota* (2% ± 2.83%), while *Desulfobacterota* (15.5% ± 2.26%) remained constant in the gut microbiota of male subjects. In female mice, by contrast, a reduction in *Bacteriodetes* (21% ± 29.7%) and *Desulfobacterota* (13.05% ± 5.16%) was observed, with a two-fold increase in *Firmicutes* (65.95% ± 34.86%) (Figure 12).

**Figure 13:**
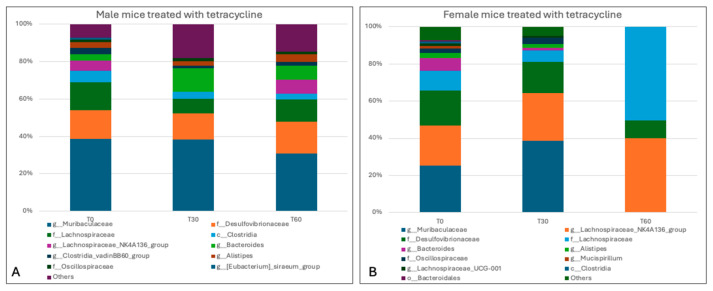
Taxa bar plot showing the relative abundance of the genera identified in male (**A**) and female mice (**B**) treated with tetracycline at the different experimental times (0, 30, and 60 days).

At the genus level, moreover, tetracycline treatment in male mice determined a decrease in *Lachnospiraceae* (10.05% ± 2.76%), *Lachnospiraceae_NK4A136_group* (3.65% ± 5.16%), *Clostridia_vadin BB60_group* (1.7% ± 0.42%), and *Muribaculaceae* (34.55% ± 5.30%), and an increase in *Bacteroides* (10.1% ± 3.39%). In the female group, a decrease was observed in the genera belonging to the phyla *Bacteroides*, i.e., *Muribaculaceae* (19.3% ± 27.29%), *Bacteroides* (0.8% ± 1.13%), and *Alistipes* (0.9% ± 1.27%), and a considerable increase in the genera belonging to the phyla *Firmicutes*: *Lachnospiraceae_NK4A136_group* (33.05% ± 10.11) and *Lachnospiraceae* (28.25% ± 31.47%) (Figure 13).

Overall, the results obtained from the analysis of bacterial species in the present study showed that chlortetracycline treatment can significantly impact on the composition of the gut microbiota, causing dysbiosis, especially in females.

### 3.6. Histopathological and Immunohistochemical Analysis

Histological examination of livers yielded the following results in the control group: 21.4% (3/14) scored as grade 0; 64.3% (9/14) as grade 1; 7.1% (1/14) as grade 2; and 7.1% (1/14) as grade 3. The group pretreated for 4 weeks with tetracyclines showed hepatic degeneration as follows: 35.7% (5/14) scored as grade 0; 35.7% (5/14) as grade 1; 7.1% (1/14) as grade 2; and 21.4% (3/14) as grade 3. In the group pretreated for 4 weeks with sulfonamides, 33.3% (4/12) were scored as grade 0; 33.3% (4/12) as grade 1; 8.33% (1/12) as grade 2; and 25% (3/12) as grade 3.

**Figure 14:**
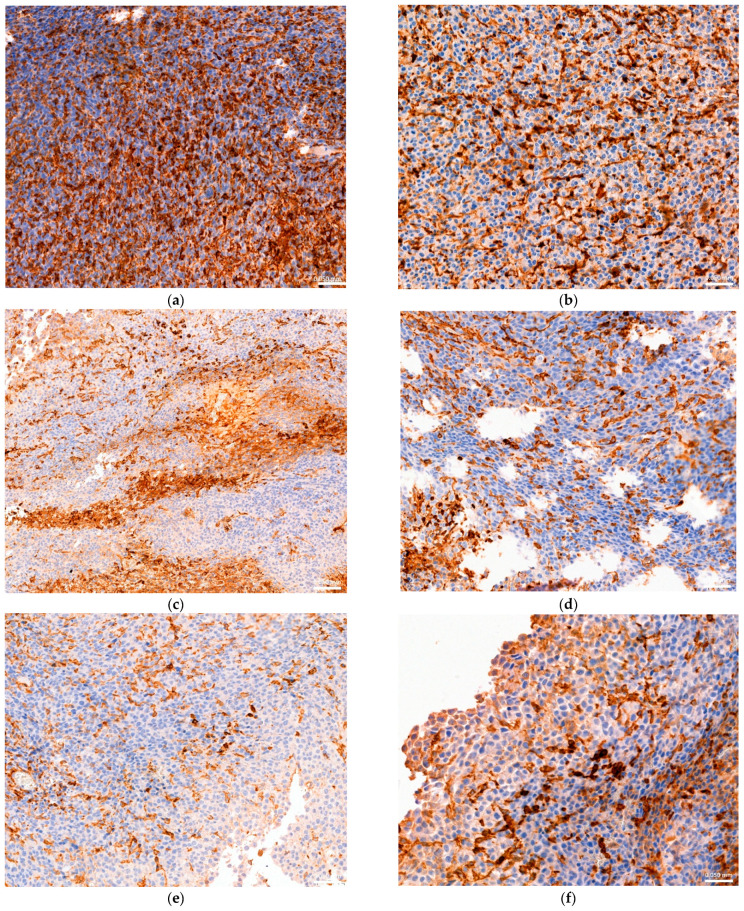
Immunohistochemical pictures of subcutaneous tumor samples with C4 and CD8 cytoplasmic and perinuclear expression (20x). Control group immunostained CD4 (**a**) and CD8 (**b**); group treated with sulfonamides immunostained CD4 (**c**) and CD8 (**d**); group treated with tetracyclines immunostained CD4 (**e**) and CD8 (**f**).

Immunohistochemical results are summarized in Figure 14, showing cytoplasmic and perinuclear expression. In the control group, the analysis showed minimum and maximum mean values of 4.5 and 22.9 for CD4 and 4.7 and 28.9 for CD8, respectively. In the group pretreated with sulfonamides, the minimum and maximum mean values were 5.3 and 19.6 for CD4 and 4.9 and 21 for CD8, respectively.

In the group pretreated with tetracyclines, the minimum and maximum mean values were 2.3 and 9.8 for CD4 and 2.7 and 11.4 for CD8 (Table 9).

## 4. Discussion

The “One health” approach regards antimicrobial resistance as a major challenge. The data obtained during the different phases of the present study, which included in vivo experimentation, microbiome analysis, and immunohistochemical analysis, showed a different response to the two antimicrobials used in comparison with control mice. The two subpopulations of T cells detected, CD4 and CD8, play different roles in antitumour immunity. Indeed, CD4 cells, which are known as T-helper cells, could potentially be involved in the first phases of antitumoral immune responses, though their role is not yet well understood. By contrast, CD8 cells are known as cytotoxic lymphocytes, and are involved in the surveillance, recognition, and killing of malignant cells [39]. Immunohistochemical examination performed on tumor samples revealed a significant expression of both T cell populations in the control group (Figure 14a,b), while their values were moderately lower in the group treated with sulfonamides (Figure 14c,d). The expression of CD4 and CD8 was even lower in the group treated with tetracyclines (Figure 14e,f). It can therefore be deduced that the use of tetracyclines at the human-equivalent dose (HED) substantially affects the immune response. These data suggest that treatment with antimicrobial drugs may reduce the activity of the immune system and its ability to react properly to the proliferation of tumor cells [40].

In addition, alteration of the gut microbiota appears to be an important risk factor for the development of colorectal cancer. Dysbiosis appears to play a crucial role in these mechanisms. Previous studies have established that antimicrobials can cause dysbiosis, leading to an imbalance in gut microbial populations and a reduction in microbial diversity. This dysbiosis is a key factor in promoting pathological changes, including carcinogenesis. Our results revealed a significant alteration to the gut microbiota composition in antimicrobial-treated mice, with marked differences in bacterial taxa known to contribute to immune modulation and cancer progression. In particular, in female antimicrobial-treated mice, we observed a variation of the Firmicutes/Bacteroidetes ratio, which is a major marker of the dysbiosis that facilitates intestinal inflammation. The persistence of this condition causes chronic inflammation of the intestine, leading to tissue damage and repair, which is associated with an increased incidence of colorectal cancer [41].

Our in vitro experiments with the CT26 colon adenocarcinoma cell line did not show significant effects of the antimicrobials on cell viability, while the in vivo experiments revealed a clear impact on tumor growth, particularly in female mice. These findings highlight the complexity of interactions among antimicrobials, the gut microbiota, and the host immune response. The absence of in vitro effects suggests that the microenvironment, which is influenced by the microbiota, plays a crucial role in modulating tumor growth, and that this effect is not replicable in a monoculture system. In addition, the in vivo experiments revealed a significant sex-dependent difference in tumor growth rates. Female mice treated with chlortetracycline exhibited greater tumor growth than males and control mice. This observation is consistent with the literature, which highlights the role of estrogens in promoting tumor growth [42]. Indeed, estrogens can enhance the secretion of factors that favor tumor progression, and this effect may be exacerbated by antimicrobial-induced dysbiosis, which could further modulate the immune response and inflammatory pathways. By contrast, sulfonamide-treated animals did not show a statistically significant increase in tumor growth in comparison with controls, suggesting a less marked impact of this antimicrobial class on tumorigenesis. However, on comparing the two treated groups, tetracycline had a more substantial effect on tumor promotion, indicating that different classes of antimicrobials may have varying impacts on cancer risk, possibly owing to their differential effects on the microbiota.

These findings may have important implications for public health and food safety. The presence of antimicrobial residues in food products, even within regulatory limits, could pose a risk for cancer development through mechanisms involving alterations of the gut microbiota and modulation of the immune system. The data suggest that there may be a need to revise current safety limits, in order to consider long-term exposure risks and the potential for sex-specific effects. These results underscore the importance of continued monitoring and regulation of antimicrobial use in veterinary medicine, as well as the need for further research into the long-term effects of low-dose antimicrobial exposure through diet.

## 5. Conclusions

This study highlights the significant impact that prolonged exposure to antimicrobial residues, particularly tetracyclines, can have on the gut microbiota and tumor progression. The results suggest that, even at concentrations deemed safe by current regulations, these antimicrobials can promote cancer growth, with a pronounced effect in female subjects. These findings emphasize the need for a re-evaluation of current food safety standards concerning antimicrobial residues and suggest that a more nuanced approach may be necessary in order to better protect public health. Future research should focus on elucidating the mechanisms underlying these sex-dependent differences and the specific roles of various gut microbiota populations in modulating cancer risk. Additionally, there is a need to explore alternative strategies in animal husbandry in order to minimize antimicrobial use, thus reducing the potential health risks associated with antimicrobial residues in the human diet.

## Figures and Tables

**Table 1:** Extract Reg 37/2010 for chlortetracycline and sulfonamides.

Pharmacologically Active Substance	Marker Residue	Animals Species	MRL	Target Tissues	Other Provisions	Therapeutic Classification
Chlortetracycline	Sum of parent drug and its 4-epimer	All food-producing species	100 μg/kg 300 μg/kg 600 μg/kg 100 μg/kg 200 μg/kg	Muscle Liver Kidney Milk Eggs	For fin fish, the muscle MRL relates to ‘muscle and skin in natural proportions’. MRLs for liver and kidney do not apply to fin fish.	Anti-infectious agents/Antimicrobials
Sulfonamides (all substances belonging to the sulfonamide group)	Parent drug	All food-producing species	100 μg/kg 100 μg/kg 100 μg/kg 100 μg/kg	Muscle Fat Liver Kidney	The combined total residues of all substances within the sulfonamide group should not exceed 100 μg/kg. For fin fish, the muscle MRL relates to ‘muscle and skin in natural proportions’. MRLs for fat, liver, and kidney do not apply to fin fish. Not for use in animals from which eggs are produced for human consumption.	Anti-infectious agents/Chemotherapeutics/

**Table 2:** Groups of recruited animals.

Mice	Groups
Male	Control Treatment with sulfonamides Treatment with tetracyclines
Female	Control Treatment with sulfonamides Treatment with tetracyclines

**Table 3:** Food matrices considered and the related limits imposed by Reg. 37/2010.

Matrix	Chlortetracycline	Sulfonamides
Muscle (beef, pork, poultry, fish)	100 µg/kg	100 µg/kg
Cow’s milk	100 µg/kg	100 µg/kg
Chicken eggs (for chlortetracycline only)	200 µg/kg	-

**Table 4:** Protein intake for the diet assumed for research purposes.

Food	Portion	Protein Content	Total (Per Portion)
Milk	200 g	3.5 g	7 g
Egg	1 (53 g CREA data)	12.4 g	6.572 g
Meat	100 g	23.3 g	23.3 g
Fish	100 g	17 g	17 g
			
Total	453 g of food		53.872 g of protein

**Table 5:** Calculation of hypothetical antimicrobial intake.

Food	Portion	Chlortetracycline (Limit Reg. 37/2010)	Total Chlortetracycline	Sulfonamides (Limit Reg. 37/2010)	Total Sulfonamides	Total Antimicrobial Residues
Milk	200 g	100 µg/kg	(100 × 0.2) = 20 µg	100 µg/kg	(100 × 0.2) = 20 µg	40 µg
Eggs	1 (53 g, CREA date)	200 µg/kg	(200 × 0.053) = 10.6 µg	-	-	10.6 µg
Meat	100 g	100 µg/kg	(100 × 0.1) = 10 µg	100 µg/kg	(100 × 0.1) = 10 µg	20 µg
Fish	100 g	100 µg/kg	(100 × 0.1) = 10 µg	100 µg/kg	(100 × 0.1) = 10 µg	20 µg
						
Total	453 g of food	-	50.6 µg	-	40 µg	90.6 µg

**Table 6:** Calculation of the amount of antimicrobial per kg of body weight.

Substance	Total Substance Meal/Day	Weight	Substance/kg Body Weight
Chlortetracycline	50.6 µg	60 kg	(50.6/60) = 0.84 µg/kg
Sulfonamides	40 µg	60 kg	(40/60) = 0.67 µg/kg

**Table 7:** The percentage of rooting in females.

	Days	11	14	17
% rooting females	Controls	14.2	14.2	100
	Chlortetracycline	42.8	71.4	100
	Sulfonamides	42.8	57.1	90

**Table 8:** The percentage of rooting in males.

	Days	13	16	18
% rooting males	Controls	14.3	57	57.1
	Chlortetracycline	0	42.9	42.9
	Sulfonamides	0	42.9	57.1

**Table 9:** N = number of individuals per group. The minimum and maximum values refer to the mean value.

		CD4+		CD8+	
Groups	N	Minimum	Maximum	Minimum	Maximum
Control	14	4.5	22.9	4.7	28.9
Sulfonamides	12	5.3	19.6	4.9	21
Tetracyclines	14	2.3	9.8	2.7	11.4

## Data Availability

All data used in the current study are available from the corresponding author upon reasonable request.

## Supplementary Materials

The following supporting information can be downloaded at: https://www.mdpi.com/article/10.3390/ani15030315/s1, Figure S1: Beta diversity analysis of mice microbiota for paired samples; Figure S2: Beta diversity analysis of mice microbiota for unpaired samples.
