# Supplementary material for: The Use of Antimicrobials in Animal Husbandry as a Potential Factor for the Increased Incidence of Colorectal Cancer: Food Safety and Kinetics in a Murine Model
Source: Animals (Basel). 2025 Jan 23;15(3):315. doi: 10.3390/ani15030315 (PMC11815752; doi:10.3390/ani15030315)
Supplement: Supplementary file 1 [file animals-15-00315-s001.zip › animals-3405289-supplementary.pdf]

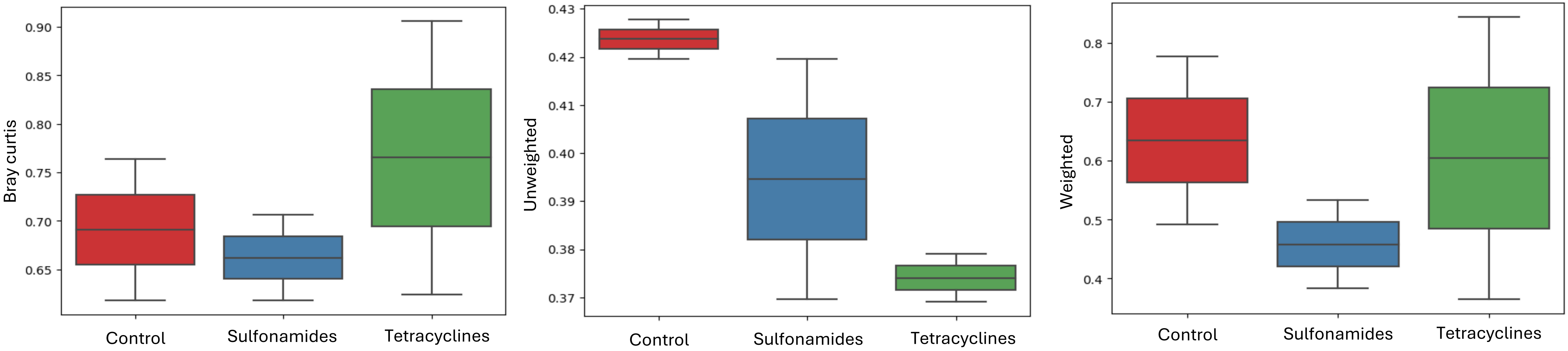

Supplementary Figure 1. Beta diversity analysis of mice microbiota for paired samples. Distance boxplots showing **(A)** Bray Curtis distance, Unweighted Unifrac **(B)** and Weighted Unifrac **(C)** for the three gropus: control (red), sulfonamides (blue) and tetracycline (green). Wilcoxon signed-rack test showed a p-value >0.05.

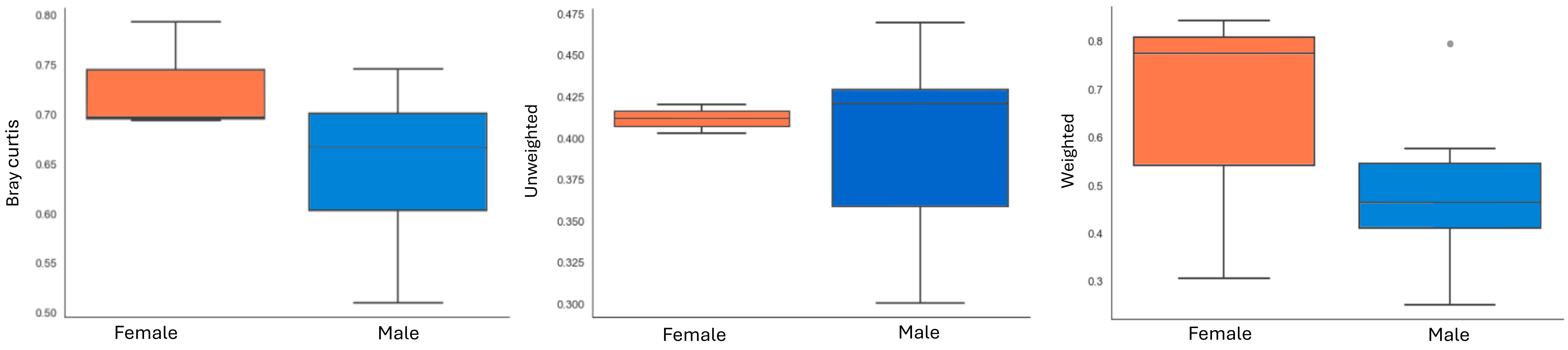

Supplementary Figure 2. Beta diversity analysis of mice microbiota for unpaired samples. Distance boxplots showing **(A)** Bray Curtis distance, Unweighted Unifrac **(B)** and Weighted Unifrac **(C)** for female mice group (orange) and male mice group (blue). Mann-Witney U test showed a p-value >0.05.
